# Heteroligand Metal Complexes with Extended Redox Properties Based on Redox-Active Chelating Ligands of o-Quinone Type and Ferrocene

**DOI:** 10.3390/molecules27123928

**Published:** 2022-06-19

**Authors:** Svetlana V. Baryshnikova, Andrey I. Poddel’sky

**Affiliations:** G.A. Razuvaev Institute of Organometallic Chemistry, Russian Academy of Sciences, 603950 Nizhny Novgorod, Russia; baryshnikova@iomc.ras.ru

**Keywords:** heteroligand complex, redox-active ligand, o-benzosemiquinone, o-aminophenolate, o-iminophenolate, metallocene, ferrocene, synthesis, redox properties, electron transfer complex

## Abstract

A combination of different types of redox-active systems in one molecule makes it possible to create coordination compounds with extended redox abilities, combining molecular and electronic structures determined by the features of intra- and intermolecular interactions between such redox-active centres. This review summarizes and analyses information from the literature, published mainly from 2000 to the present, on the methods of preparation, the molecular and electronic structure of mixed-ligand coordination compounds based on redox-active ligands of the o-benzoquinone type and ferrocenes, ferrocene-containing ligands, the features of their redox properties, and some chemical behaviour.

## 1. Introduction

Currently, the coordination chemistry of redox-active compounds is attracting a lot of attention from various research groups worldwide [1,2,3,4,5,6,7,8,9,10,11,12,13,14]. Such growing attention is caused by the fact that unlike “classical” ligands, redox-active ones significantly expand the range of redox transitions in their complexes and the number of different electronic states associated with combinations of oxidized (reduced) forms of redox-active ligands and the central metal atom.

Distinguished representatives of this type of ligands are o-benzoquinones and related O,N-; N,N′-; S,N-; etc. chelating ligands [15,16,17,18,19,20,21,22,23,24,25]. They can reversibly accept one or two electrons while in the coordination sphere of the metal, thus forming the radical anion or dianion form of the ligand. In turn, this property of redox-active ligands allows their complexes to be active in various oxidative addition, reductive elimination, and some other reactions, which are in many respects the key to most catalytic processes [26,27,28,29,30]. Some catalytic systems for various reactions of organic chemistry based on complexes with redox-active ligands of this type have been created to date, e.g., hydrophosphination, hydroarylation, hydroamination [31], formation of C–C bonds [32,33,34,35], C–O bonds [36], C–N bonds [37,38], activation of C–Hal bonds [39], etc.; catalysts for the oxidation of various substrates, including catechol oxidase, etc. [40,41,42,43]; catalysts of polymerization [44,45], hydroboronation, and cyanosilylation [46], etc.

On the other hand, the chemistry of ferrocene has been actively developed in recent years [47,48,49,50,51]. The unique geometry of the sandwich structure, as well as the ability to perform reversible oxidation, forming a ferrocene/ferrocenium (Cp_2_Fe/Cp_2_Fe^+^) redox pair, makes it a suitable object for studying electron transfer processes, utilising ferrocenes in the design of molecular magnets [52,53,54], in catalysis [55,56,57], and as a standard and as electrochemical agents in electrochemical studies [58]. Functionalized ferrocene derivatives are widely used in coordination chemistry as donor ligands [59,60,61].

A combination of different types of redox-active systems (e.g., derivatives of the quinone series and ferrocene) in one molecule makes it possible to develop coordination compounds with extended redox capabilities, combining molecular and electronic structures determined by the features of intra- and intermolecular interactions between these redox-active centres. Thus, the study of metal complexes that combine various redox centres, such as o-benzoquinone/o-iminobenzoquinone and ferrocene, is of undoubted interest from the point of view of the formation of new types of coordination compounds that can be involved in a wide range of redox processes. This makes it possible to reveal the features of the formation of radical ion pairs and the factors that control the process of electron transfer. However, to date, not many works are devoted to studying systems based on heteroligand metal complexes that simultaneously contain several redox-active centres, such as o-benzoquinone-related ligands and the ferrocene group in the literature. In the present review, we summarize the literature dealing with the coordination chemistry of systems combining redox-active ligands of o-quinone, o-iminoquinone, and related types with ferrocene- or ferrocenyl-containing ligands. The list of complexes covered in this review is given in Appendix A.

## 2. Metal Complexes Based on Redox-Active Ligands in Reactions with Ferrocenes

**Scheme 1:**
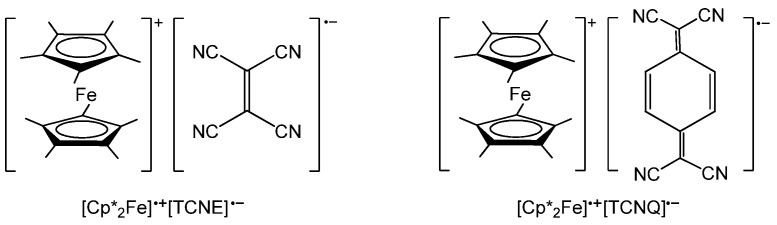
The charge-transfer systems based on decamethylferrocene and tetracyanoethylene (TCNE) or 7,7,8,8-tetracyano-p-quinodimethane (TCNQ).

The ability of ferrocene to perform reversible oxidation makes it possible to create charge-transfer systems with ferrocene acting as an electron donor [62,63,64,65,66]. The salts based on decamethylferrocene Cp*_2_Fe and tetracyanoethylene (TCNE) or 7,7,8,8-tetracyano-p-quinodimethane (TCNQ) were the first examples of such systems (Scheme 1). Miller and co-workers [67] found that the unit cell contains linear chains of alternating [Cp*_2_Fe]^•+^ radical cations and TCNE^•−^ radical anions with spin S = 1/2 (Cp* = pentamethylcyclopentadienyl). For these compounds, the authors have found a ferromagnetic exchange between the donor (Cp*_2_Fe) and the acceptor (TCNE or TCNQ) in the linear chain …D^•+^A^•−^D^•+^A^•−^D^•+^A^•−^… at low temperatures [62].

**Scheme 2:**
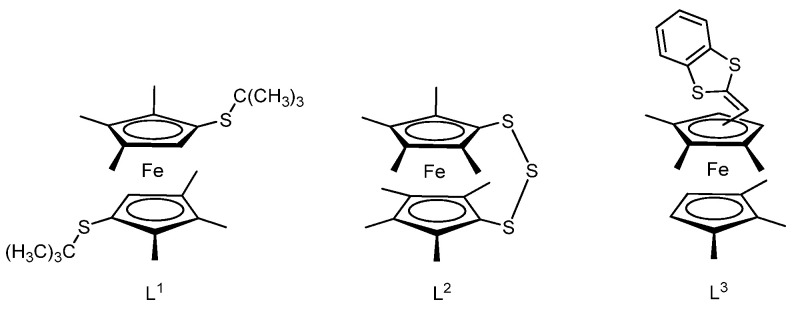
Hexamethyl-substituted ferrocene derivatives L^1^–L^3^ containing thioether, S-heterocyclic, or vinyl tetrathiafulvalene substituents.

Other examples of charge-transfer systems are complexes based on hexamethyl-substituted ferrocene derivatives, for example, L^1^–L^3^ containing thioether, S-heterocyclic, or vinyl tetrathiafulvalene substituents (Scheme 2) [68].

**Scheme 3:**
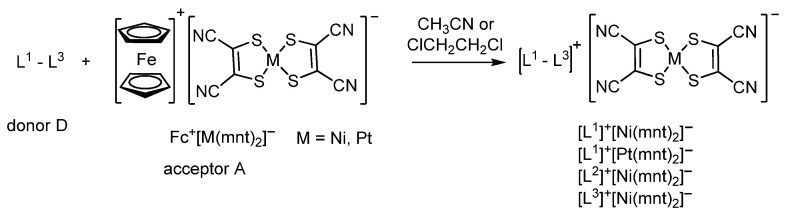
The formation of charge-transfer complexes0 [L^1–3^]^+^[M(mnt)_2_]^−^ (M = Ni, Pt).

Acting as electron donors (D), such ligands can easily be oxidized with electron acceptors (A) to form paramagnetic salts containing nickel or platinum(III) dimers (Scheme 3). The authors have shown that in the crystal packing of charge-transfer complexes (CT complexes) [L^2^]^+^[Ni(mnt)_2_]^−^ and [L^3^]^+^[Ni(mnt)_2_]^−^, the donor and acceptor fragments are arranged in the order D^+^A^–^A^–^D^+^ with different arrangements of dimers [{Ni(mnt)_2_}_2_]^2−^, which leads to a strong antiferromagnetic exchange between paramagnetic centres in A^−^A^−^ fragments (J = −302 cm^−1^ for [L^2^]^+^[Ni(mnt)_2_]^−^, and J = −630 cm^−1^ for [L^3^]^+^[Ni(mnt)_2_]^−^).

In contrast to these complexes, salts [L^1^]^+^[Ni(mnt)_2_]^−^ and [L^1^]^+^[Pt(mnt)_2_]^−^ demonstrate structural ordering D^+^A^−^D^+^A^−^ and ferromagnetic nature of the exchange between [{Ni(mnt)_2_}_2_]^2−^ dimers at low temperature. Throughout the CT complexes series, the contribution of the magnetic exchange interaction between the donor (D^+^) and acceptor (A^−^) does not significantly affect the magnetic susceptibility of these ionic systems [68].

**Scheme 4:**
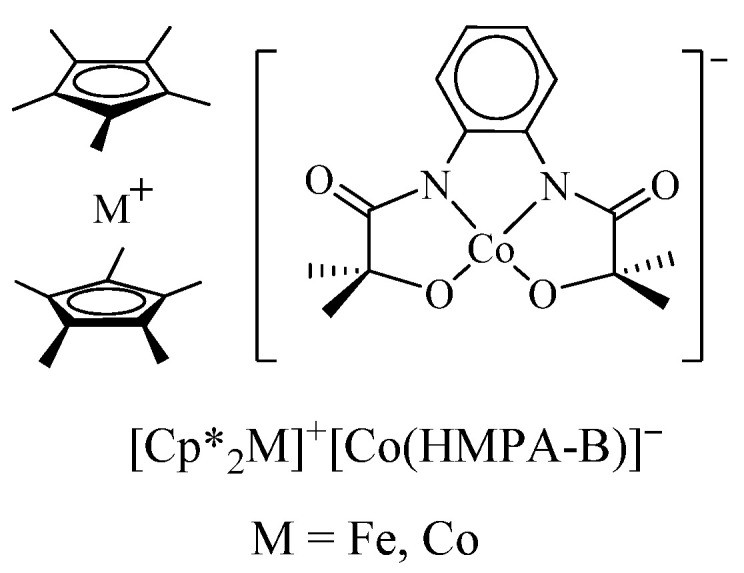
Charge-transfer complexes [Cp*_2_M]^+^[Co(HMPA-B)]^−^ based on metallocenes Cp*_2_M (M = Fe, Co) and cobalt(III) bis-amidophenolate.

B.M. Hoffman et al. [69] have characterized charge-transfer complexes [Cp*_2_M]^+^[Co(HMPA-B)]^−^ based on metallocenes Cp*_2_M (M = Fe, Co) and cobalt(III) bis-amidophenolate complexes (Scheme 4). X-ray diffraction analysis has shown the formation of a stacked structure in [Cp*_2_Fe]^+^[Co(HMPA-B)]^−^. The alternation of the [Cp*_2_Fe]^+^ fragment with the plane of the [Co(HMPA-B)]^−^ anion of the complex was observed. This structure is reflected in the magnetic properties of the compound: a ferromagnetic exchange was found between the [Cp*_2_Fe]^+^ cation with spin S = 1/2 and the complex anion inside one stack.

**Scheme 5:**
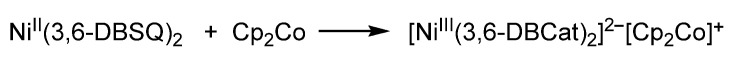
Reaction of nickel(II) bis-o-benzosemiquinolate with cobaltocene.

An analysis of literature data on metal complexes based on redox-active quinone-type ligands showed that this class of compounds could also react with metallocenes (usually cobaltocene, and only a few examples of ferrocene) acting as reducing agents and form ionic-type complexes [70,71,72]. C.G. Pierpont et al. [70] have shown that the interaction of cobaltocene and nickel(II) bis-o-benzosemiquinone complexes Ni^II^(3,6-DBSQ)_2_ proceeds as the reduction of both o-benzosemiquinone ligands to catecholates along with the oxidation of the central metal atom Ni(II) to Ni(III) (Scheme 5, 3,6-DBSQ is a radical anion 3,6-di-tert-butyl-o-benzosemiquinone, 3,6-DBCat is a dianion 3,6-di-tert-butylcatecholate).

**Scheme 6:**
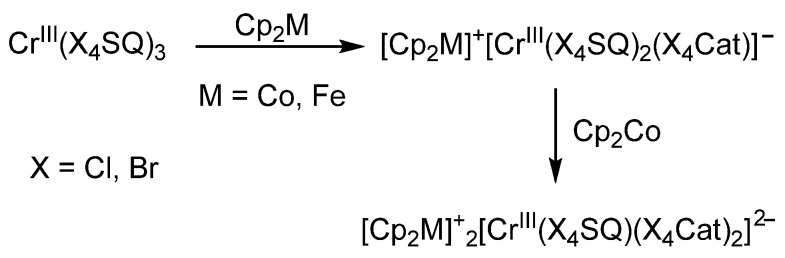
Reaction of chromium(III) tris-o-benzosemiquinolate with ferrocene and cobaltocene.

S. Kitagawa et al. [71,72] characterized a series of related ionic chromium complexes in sufficient detail. These compounds were prepared by the reaction of ferrocene or cobaltocene with chromium(III) tris-o-benzosemiquinones Cr^III^(X_4_SQ)_3_, where X = Cl or Br (Cl_4_SQ is tetrachloro-o-benzosemiquinone, Br_4_SQ is tetrabromo-o-benzosemiquinone) (Scheme 6). The authors pay attention to the fact that chromium(III) monoanionic complexes can be synthesized by reducing the initial complex with cobaltocene or ferrocene. In contrast, the dianionic complex derivatives can be isolated only by the interaction of chromium tris-o-benzosemiquinolate with cobaltocene in a molar ratio of 1:2. Of course, this is due to the significant difference in the redox potentials of the selected metallocenes. For the mono- and di-reduced forms, in the electronic absorption spectra, charge-transfer bands between ligands in different oxidation states are observed in the range of 3130–10,000 cm^−1^.

**Scheme 7:**
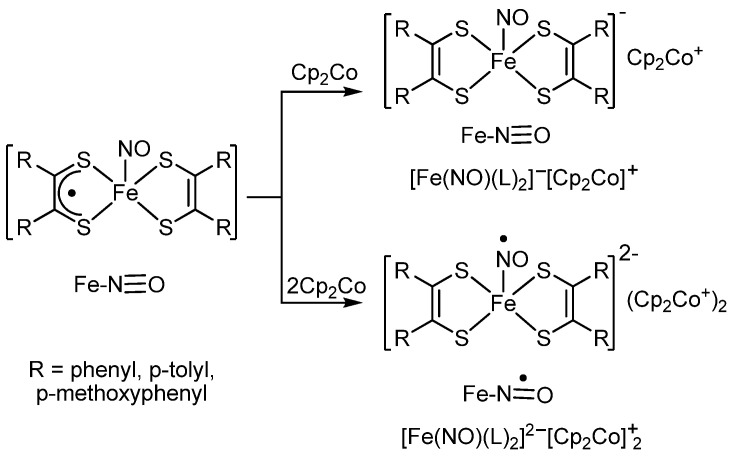
The reduction of mixed-ligand iron nitrosyl complex with mono- and di-reduced forms of S,S′-dithiolene ligands with cobaltocene.

Besides o-quinone complexes, other types of complexes with chelating redox-active ligands also undergo the reduction of redox-active ligand with cobaltocene. For example, K. Wieghardt and co-authors [73] have shown that the interaction of one equivalent of cobaltocene with iron nitrosyl complex with mono- and di-reduced forms of S,S′-dithiolene ligands results in the reduction of the radical anion ligand to a dianion ligand. In turn, the addition of another equivalent of cobaltocene also caused a reduction of nitrosyl ligand (Scheme 7).

**Scheme 8:**
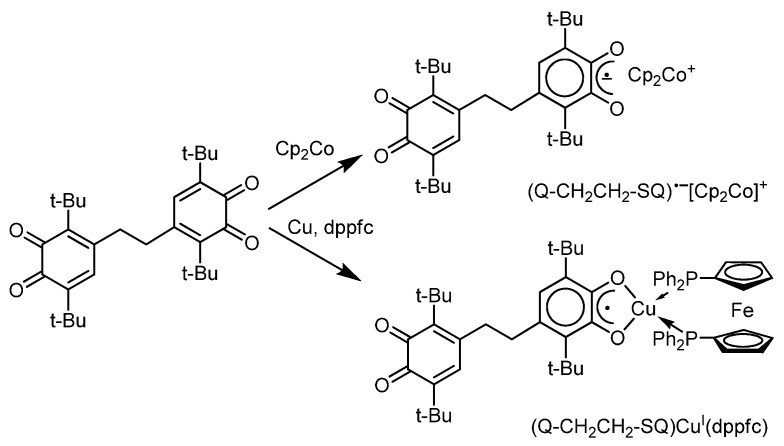
The reduction of di-o-benzoquinone Q-CH_2_-CH_2_-Q with cobaltocene or elementary copper in the presence of ferrocene containing neutral diphosphine ligand dppFc.

The fact of the formation of the monoanionic derivative [Fe(NO)(L)_2_]^−^[Cp_2_Co]^+^ is confirmed by X-ray diffraction data: the authors have unequivocally indicated the formation of a complex with chelate-bound fragments corresponding to the dianion form of the dithiolene ligand.

o-Semiquinone complexes with a doublet ground spin state are fruitful objects for EPR spectroscopic investigations. An inalienable property of o-quinones is the possibility of forming reduced o-semiquinone derivatives with different reductants, such as alkali metals, cobaltocene, or copper in the presence of some donor ligands, e.g., phosphines [74,75,76,77]. Thus, G.A. Abakumov et al. [74] have shown that cobaltocene efficiently acts as a reducing agent for di-o-benzoquinone, forming the derivative (Q-CH_2_CH_2_-SQ)^•−^[Cp_2_Co]^+^ (Scheme 8). The isotropic EPR spectrum of this radical anion complex is a doublet of triplets (1:2:1) due to the interaction of an unpaired electron with a proton of the o-semiquinone ring and two protons of the methylene group closest to it with hyperfine coupling (HFC) constants a_i_(^1^H_SQ_) = 3.6 G and a_i_(2 ^1^H_CH2_) = 2.1 G, respectively.

Using 1,1′-bis-(diphenylphosphino)ferrocene (dppfc) as an example, the authors have shown that ferrocenes with donor phosphorus-containing groups could act as neutral donor ligands in o-benzosemiquinone complexes, e.g., (Q-CH_2_CH_2_-SQ)Cu^I^(dppfc) (Scheme 8) [74]. The EPR spectrum of this copper(I)-diphosphine complex reflects the HFC of an unpaired electron with a proton of the o-benzosemiquinone moiety (with HFC constants a_i_(^1^H_SQ_) = 3.2 G and a_i_(2 ^1^H_CH2_) = 2.0 G), magnetic isotopes of a copper atom, and two phosphorus atoms (a_i_(2 ^31^P) = 18.9 G). Interestingly, only components corresponding to the transition Δm_s_(^31^P) = ±1 are observed at room temperature. In contrast, as the temperature rises to 350 K, the intensity of the components Δm_s_(^31^P) = 0 increases, which at 300 K are significantly broadened and have zero intensity. The authors attribute this effect to the slow exchange of phosphine ligands between the apical and equatorial positions in the copper coordination sphere on the EPR time scale. It is noteworthy that dppFc was one of the widely applied ligands in the chemistry of mixed-ligand bis-phosphino-copper(I) o-semiquinones (e.g., (3,6-DBSQ)Cu^I^(dppfc) [75], Ph_3_Sb(Cat-SQ)Cu^I^(dppfc) [76], (Q-TTF-SQ)Cu^I^(dppfc) [77].

**Scheme 9:**
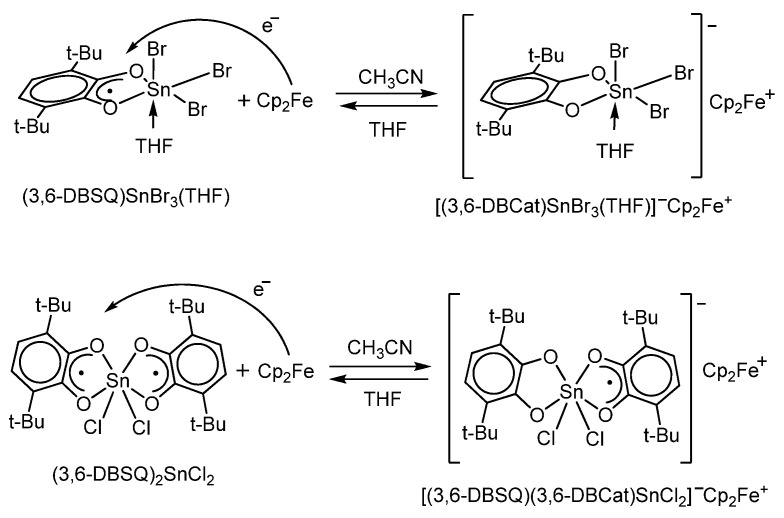
The reduction of mono- and di-o-benzosemiquinonato tin(IV) halides with ferrocene to give charge-transfer complexes.

In [78], the first example of charge transfer in systems containing a metallocene and a non-transition metal complex with redox-active o-quinone-type ligands was described. G.A. Abakumov et al. have shown that the interaction of ferrocene with mono- and bis-3,6-di-tert-butyl-o-benzosemiquinolate halide-containing tin(IV) complexes in acetonitrile leads to a reversible electron transfer from ferrocene to the o-benzosemiquinone ligand and the formation of ionic complexes [(3,6-DBCat)SnBr_3_(THF)]^−^[Cp_2_Fe]^+^ and [(3,6-DBSQ)(3,6-DBCat)SnCl_2_]^−^[Cp_2_Fe]^+^ (Scheme 9). This interaction between ferrocene and halogenated Sn(IV) o-benzosemquinolates depends on the nature of the solvent: the replacement of acetonitrile with tetrahydrofuran (THF) shifts the equilibrium shown in Scheme 9 to the starting reagents. The formation of ionic complexes was confirmed by UV, UV-Vis, and EPR-spectroscopy as well as X-ray diffraction. For example, the electron transfer from Cp_2_Fe to (3,6-DBSQ)SnBr_3_(THF) or (3,6-DBSQ)_2_SnCl_2_ results in the appearance of new absorptions with maxima at about 620 nm typical for the complexes containing a ferrocenium cation. Additionally, a broad ligand-to-ligand charge-transfer band (LLCT) with a maximum at 930 nm was observed in the UV-spectrum of [(3,6-DBSQ)(3,6-DBCat)SnCl_2_]^−^[Cp_2_Fe]^+^ in acetonitrile. This LLCT band is typical for complexes containing mixed-valency ligands (in this case, SQ and Cat).

Magnetochemical studies of the mixed-ligand complex [(3,6-DBSQ)(3,6-DBCat)SnCl_2_]^−^[Cp_2_Fe]^+^ containing the ferrocenium radical cation (D^•+^) and the radical anion of the tin(IV) complex (A^•−^) have shown that complexes of this type demonstrate ferromagnetic exchange in a linear chain of alternating donor-acceptor-donor fragments (···D^+•^A^−•^D^+•^A^−•^···).

The oxidation of ferrocene with tin(IV) o-semiquinone complexes is quite unusual in the chemistry of o-semiquinone complexes. Usually, ferrocenium salts are used as a one-electron oxidant to turn catecholato complex into an o-semiquinone derivative.

## 3. Metal Complexes Based on Ferrocene-Containing Redox-Active Ligands

Much attention is paid to the study of reactions with electron transfer, which are of crucial importance in many fundamental catalytic and biochemical processes, including photosynthesis, respiration, the transmission of nerve impulses, etc. [79,80,81,82,83,84,85]. Among the model systems studied, ferrocene derivatives and ferrocene-containing complexes occupy a special place [47]. As shown above, when interacting with acceptors, ferrocene forms a stable and reversible Fe(II)/Fe(III) electrochemical pair, which allows it to enter into electronic interactions with other π-systems. The resulting oxidation product is the ferrocenium cation, the paramagnetic centre, which can be involved in intra- and intermolecular magnetic exchange. From this point of view, it is interesting to consider the molecular and electronic structure, as well as the properties of various types of metal complexes: (1) compounds containing redox-active centres of different nature, such as quinone, iminoquinone, iminophenol, dithiolene, and other types of ligands substituted with a ferrocenyl group and (2) compounds with redox-active ligands coordinated to the central metal atom in a complex containing a ferrocene fragment in an additional ligand.

An analysis of the literature on the chemistry of ferrocenes has shown that one of the most common methods for the functionalization of complexes of various metals with ferrocene groups is the use of Schiff base-type ligands bound to ferrocene [86,87,88,89,90,91,92,93,94,95,96,97,98,99].

**Scheme 10:**
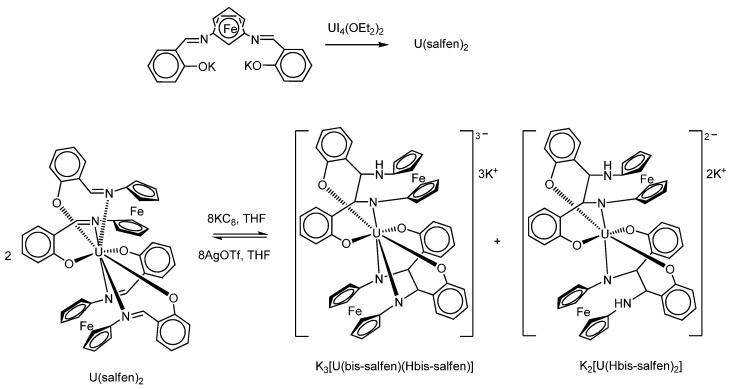
The metathesis reaction between UI_4_(OEt_2_)_2_ and potassium salt of the tetradentate Schiff base ligand containing a ferrocene group to obtain a bis-ligand uranium(IV) complex U(salfen)_2_, and its reduction.

For example, the scientific group of M. Mazzanti used the metathesis reaction between UI_4_(OEt_2_)_2_ and two equivalents of the potassium salt of the tetradentate Schiff base ligand containing a ferrocene group to obtain a bis-ligand uranium(IV) complex U(salfen)_2_ (Scheme 10) [86].

The authors have also shown that the reduction of the U(salfen)_2_ complex occurs at the ligand centre and includes the reduction of imino groups of Schiff base ligands. As a result, an intramolecular C–C bond formation occurs within the ligand between two imino groups of the ferrocene-containing ligand, but not the formation of an interligand C–C bond, as previously reported for tridentate and tetradentate Schiff bases in similar complexes without a ferrocenyl function [100,101]. The authors attribute this feature to the high flexibility of the ferrocene bridge connecting two parts of the imino groups of one ligand. 

**Scheme 11:**
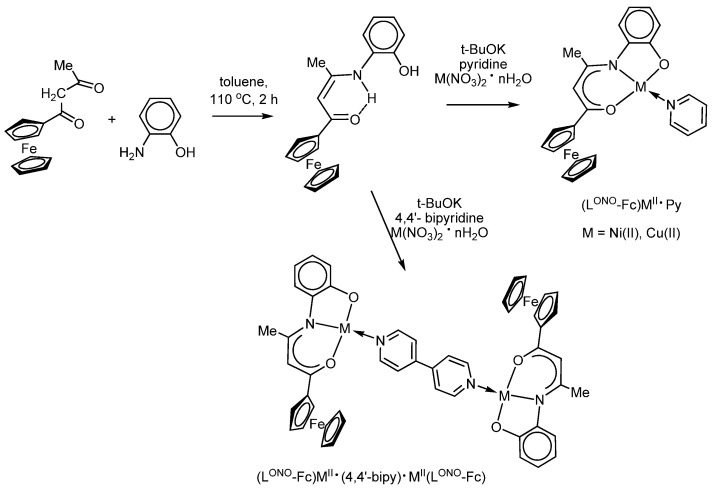
The synthesis of complexes (L^ONO^-Fc)M^II^∙Py and (L^ONO^-Fc)M^II^∙(4,4′-bipy)∙M^II^(L^ONO^-Fc), where M = Ni or Cu.

J.-R. Hamon et al. [87,89] used the condensation reaction of 2-aminophenol and the corresponding ferrocenylacetone to obtain and characterize (using IR-, ^1^H and ^13^C NMR-, UV-spectroscopy, XRD) ferrocene-containing phenol L^ONO^H_2_, which reacts readily with copper(II) and nickel(II) salts in the presence of neutral donor ligands (pyridine, 4,4′-bipyridyl), thus forming the corresponding O,N,O′-chelate-bound complexes (L^ONO^-Fc)M^II^∙Py and (L^ONO^-Fc)M^II^∙(4,4′-bipy)∙M^II^(L^ONO^-Fc), where M = Ni or Cu (Scheme 11). The related heterobimetallic copper(II) and nickel(II) complexes of the type (L^ONO^-Fc)M^II^∙(PyMP) (where PyMP is pyridyl 2,6-diphenylmethylenepyran) were described in 2019 by the same group [90]. Complexes (L^ONO^-Fc)M^II^∙Py and (L^ONO^-Fc)M^II^∙(PyMP) are very similar and display two quasi-reversible one-electron oxidation waves: at E_1/2_^1^ = 0.05–0.15 V (vs. Cp_2_Fe/Cp_2_Fe^+^ couple) and E_1/2_^2^ = 0.40–0.45 V. In each case, the first wave was associated with the oxidation of the ferrocenyl moiety, while the second wave was assigned to the further oxidation both at the iron and M(II) centres.

According to the electrochemical data, two ferrocene centres of symmetrical Ni(II) and Cu(II) complexes (L^ONO^-Fc)M^II^∙(4,4′-bipy)∙M^II^(L^ONO^-Fc) are oxidized at the same redox potential, which indicates the absence of their influence on each other through the conjugated 4,4′-bipyridyl bridge [87].

**Scheme 12:**
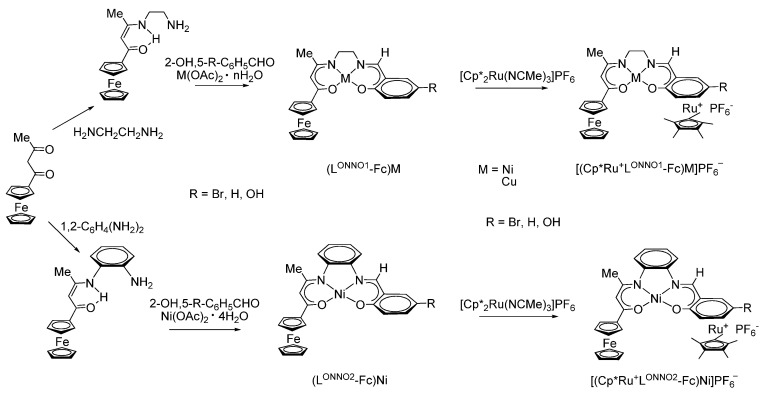
The synthesis of complexes (L^ONNOn^-Fc)M and [(Cp*Ru^+^L^ONNOn^-Fc)M]PF_6_^−^, where M = Ni or Cu (n = 1, 2).

A completely different picture is observed for a series of bi/trinuclear neutral asymmetric copper and nickel complexes based on ferrocene-containing Schiff base ligands (Scheme 12) [93,94,95].

In this case, the strong electronic interaction between two organometallic centres (Fe-Ru), namely, between the ferrocenyl group and the electron-withdrawing mixed-ligand ruthenium sandwich structure through the π-conjugated macrocyclic organometallic bridge [(L^ONNO^)Ni], is observed. The authors confirm this fact by the presence of an anodic shift in the Fe(II)/Fe(III) oxidation potential in nickel complexes by 108 and 54 mV upon passing from binuclear complexes (L^ONNO1^-Fc)Ni and (L^ONNO2^-Fc)Ni to trinuclear complexes [(Cp*Ru^+^L^ONNO1^-Fc)Ni]PF_6_^−^ and [(Cp*Ru^+^L^ONNO2^-Fc)Ni]PF_6_^−^, respectively.

Another evidence in favour of the electronic interaction between organometallic centres (ferrocene/ruthenium mixed-ligand sandwich) through the π-conjugated macrocyclic bridge was the presence of two intense broad absorption bands in the visible region of the UV spectra of solutions of complexes shown in Scheme 12 in CH_2_Cl_2_ and dimethyl sulfoxide (DMSO): a band in the range of 330–390 nm (intra-ligand π–π* transition) and a broad band in the region of 400–600 nm, which the authors attribute to the ligand-ligand and metal-ligand charge-transfer band [94]. 

**Scheme 13:**
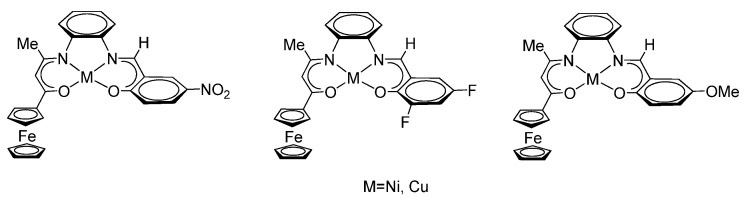
Asymmetric nickel(II) and copper(II) complexes of the type (L^ONNO^-Fe)M, M = Ni or Cu.

A series of asymmetric nickel(II) and copper(II) complexes of the related type (L^ONNO^-Fe)M [92,96], M = Ni or Cu, (Scheme 13) based on a tetradentate-bound Schiff base was synthesized by a similar method as described by the group of J.-R. Hamon [93].

Nickel and copper complexes containing electron-donor (methoxy-) and electron-withdrawing (nitro-, 3,5-difluoro-) substituents in the arene fragment of the Schiff base and a ferrocene substituent form the system D–π–D [92,96].

The interaction between the ferrocene fragment and the (L^ONNO^)M coordination centre was established according to UV spectroscopy and the electrochemical data. The authors found that nickel and copper complexes with a methoxy substituent in the arene fragment have similar oxidation potentials of 0.289 and 0.298 V, related to the reversible one-electron oxidation of the ferrocene fragment in complexes. These redox potentials are shifted to the anode region by 90 mV compared to the oxidation potential of free ferrocene. It has been shown that 5-nitro-substituted derivatives demonstrate a greater anodic shift when compared to 3,5-difluoro-substituted analogues. The fact that the oxidation of the ferrocenyl group is more difficult is explained by the influence of electron-withdrawing substituents in the metal-containing fragment based on the Schiff base [96].

Homoligand systems with ligand L^ONO^-Fc are represented by a cobalt(III) complex of the type [(L^ONO^-Fc)_2_Co]^−^[K(EtOH)_2_]^+^ [102]. The complex contains a mononuclear hexacoordinated pseudo-octahedral complex anion. The CV of this complex shows two reversible oxidation processes E_1/2_^1^ = 0.53 V (vs. Cp_2_Fe/Cp_2_Fe^+^ couple) assigned to the oxidation of ferrocenyl groups and E_1/2_^2^ = 0.69 V attributed to the oxidation of Co(III)-phenolate to Co(III)-phenoxyl derivative, while the third oxidation process at E_pa_ = 1.07 V is irreversible and presumably corresponds to the irreversible oxidation of the Co(III)-phenoxyl into unstable phenoxonium cations.

**Scheme 14:**
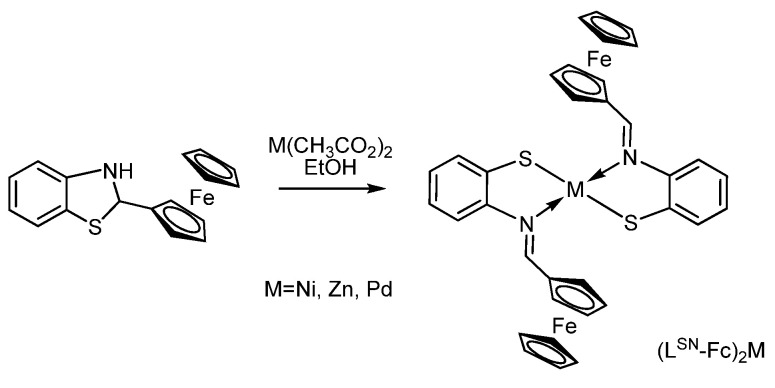
Synthesis of ferrocene-containing bis-iminothiophenolates (L^SN^-Fc)_2_M (M = Ni, Zn, or Pd).

The group of Y. Kushi has shown that ferrocene-containing bis-iminothiophenolate complexes (L^SN^-Fc)_2_M of some metals (M = Ni, Zn, and Pd) (Scheme 14) undergo two reversible one-electron oxidation processes, corresponding to the ferrocene/ferrocenium oxidation, indicating a significant interaction between the two ferrocenyl substituents [97,98].

**Scheme 15:**
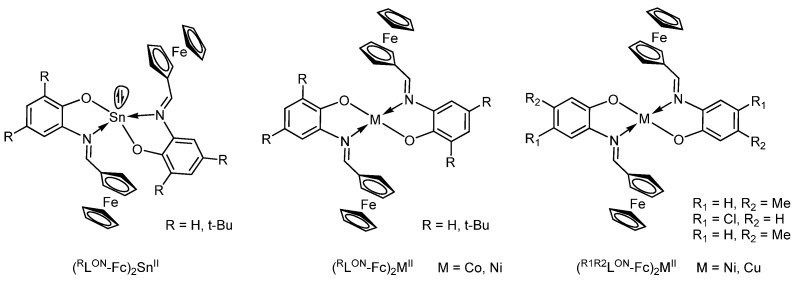
Synthesis of ferrocene-containing bis-iminophenolates (^R^L^ON^-Fc)_2_M (M = Sn, Co, or Ni), and (^R1R2^L^ON^-Fc)_2_M^II^ (M = Ni, Cu).

The related bis-ligand tin(II) complexes (^R^L^ON^-Fc)_2_Sn^II^ (Scheme 15) based on the ferrocenyl-containing O,N-chelating Schiff base ligands are described in [103]. Complexes were synthesized by the reaction of the corresponding o-iminophenol with tin(II) chloride in the presence of a base.

The related cobalt(II) and nickel(II) bis-o-iminophenolates (^R^L^ON^-Fc)_2_M^II^ (M = Co or Ni), nickel(II) and copper(II) bis-o-iminophenolates (^R1R2^L^ON^-Fc)_2_M^II^ (M = Ni or Cu) with ferrocenyl groups (Scheme 15) are reported in [104,105]. The crystalline structures of some of these complexes were established by XRD. It is noteworthy that in contrast to nickel(II) complexes which are square-planar, the aforementioned tin(II) bis-o-iminophenolates are tetragonal-pyramidal with an electron pair in the apex of pyramid [103]. The electrochemical behaviour of some complexes was investigated by means of cyclic voltammetry (CV). The CV of cobalt complex (^H^L^ON^-Fc)_2_Co in CH_2_Cl_2_ in the potential sweep from −0.5 to 1.0 V has one two-electron oxidation wave E^ox1^ = 0.8 V (vs. Ag/AgCl/KCl(sat.)) assigned to the oxidation of two ferrocenyl moieties with the generation of dicationic complexes. An increase in the scanning potential of up to 1 V·s^−1^ does not lead to the separation of the peaks, which indicates the absence of the electronic interaction between the two oxidized ferrocenyl groups. In contrast, the CVs of complexes (^tBu^L^ON^-Fc)_2_M (M = Co, Ni) with di-tert-butyl-substituted o-iminophenolato ligands contain two close waves at E^ox1^ = 0.76–0.77 V and E^ox2^ = 0.90–0.94 V corresponding to the oxidations of two ferrocenyl fragments with generation of dicationic form of complexes and the third wave at E^ox3^ = 1.37–1.38 V which was attributed to the oxidation of the coordinated phenolic fragment.

**Scheme 16:**
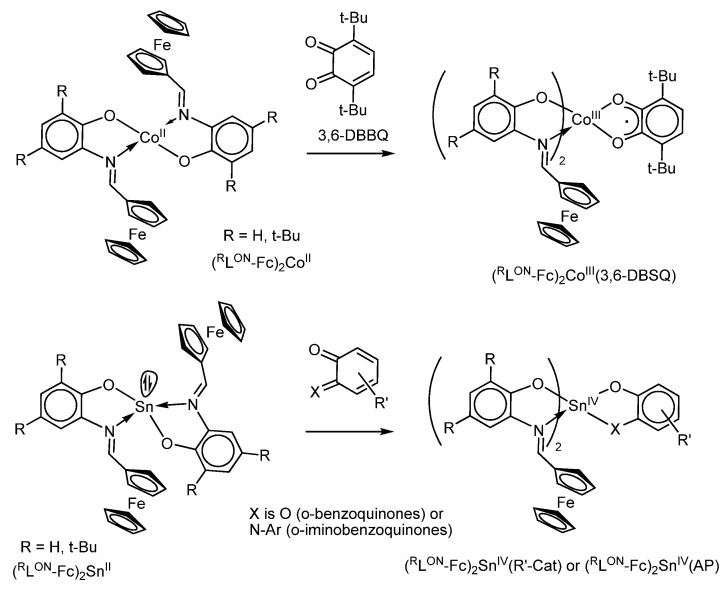
Oxidation of Co(II) and Sn(II) o-iminophenolates with o-(imino)quinones.

Cobalt(II) bis-o-iminophenolates (^R^L^ON^-Fc)_2_Co^II^ react with o-benzoquinones with a one-electron oxidation of cobalt(II) to paramagnetic cobalt(III) o-semiquinone derivatives as, for example, (^R^L^ON^-Fc)_2_Co^III^(3,6-DBSQ) (Scheme 16), which have well-resolved isotropic EPR spectra (g_i_ = 2.000-2.001, a_i_(1 ^1^H) = 2.9 G, a_i_(1 ^1^H) = 3.7 G, a_i_(^59^Co) = 11.3–11.5 G) [104].

In contrast to cobalt(II) derivatives, tin(II) bis-iminophenolates (^R^L^ON^-Fc)_2_Sn^II^ easily react with o-benzoquinones or o-iminobenzoquinones through the two-electron oxidation yielding mixed-ligand tin(IV) catecholato-bis-iminophenolates (^R^L^ON^-Fc)_2_Sn^IV^(R-Cat) (Scheme 16) or o-amidophenolato-bis-iminophenolates (^R^L^ON^-Fc)_2_Sn^IV^(AP) [106,107], where R-Cat is a substituted catecholate and AP is a substituted o-amidophenolate. These complexes undergo a series of electrochemical redox-transformations involving both ferrocene groups as well as dianionic ligand (catecholate or o-amidophenolate). The relative oxidation potentials of these redox centres depend on the acceptor properties of the redox-active chelating O,O′ or O,N ligand. An increase in the acceptor properties of redox-active o-quinonato-type ligands leads to an increase in the oxidation potentials of redox ligands as well as the following oxidation of ferrocenyl group(s). As a result, such a convergence leads to the observation of the simultaneous oxidation of the catecholate moiety and ferrocenyl group in (^H^L^ON^-Fc)_2_Sn(4,5-Cl_2_-3,6-DBCat), where 4,5-Cl_2_-3,6-DBCat is a dianion 4,5-dichloro-3,6-di-tert-butylcatecholate. These observations allow to suggest the possibility of creating mixed-ligand main-group metal complexes based on oquinone-type ligands and ferrocene capable of intramolecular electron transfer, which is well-known for transition-metal complexes.

A number of related complexes based on ferrocene-containing o-iminophenolate L^ON^-Fc^−^ (namely, (L^ON^-Fe)_2_Mn∙4H_2_O, (L^ON^-Fe)_2_VO∙H_2_O, (L^ON^-Fe)Zn(NO_3_)∙3H_2_O, and (L^ON^-Fe)Pd(CH_3_COO)∙2H_2_O) was studied for their biological activity (DNA interaction, antimicrobial, antioxidant, anticancer activities, and molecular docking) with promising results [108].

**Scheme 17:**
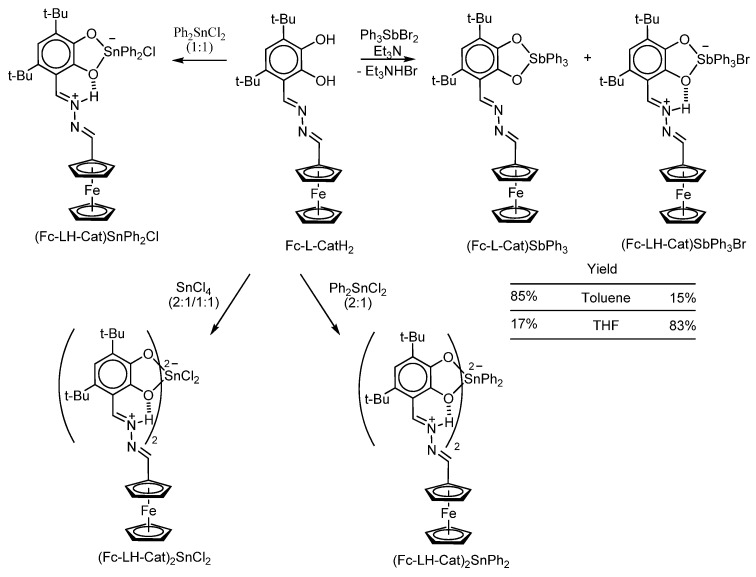
Synthesis of antimony(V) and tin(IV) complexes based on the ferrocene-containing catechol Fc-L-CatH_2_.

Some zwitterionic complexes are reported in [109]. It has been established that the reaction of Ph_3_SbBr_2_ with ferrocene-containing catechol Fc-L-CatH_2_ (Scheme 17) in the presence of a base in toluene leads to the formation of a mixture of two products with “classic” triphenylantimony(IV) catecholate (Fc-L-Cat)SbPh_3_ as the main product (85% according to ^1^H NMR spectroscopy) and the derivative (Fc-LH-Cat)SbPh_3_Br as a by-product of the reaction (with a yield of less than 15%). However, a similar reaction in THF gives the zwitterionic complex (Fc-LH-Cat)SbPh_3_Br as the main product, while the “classic” catecholate is a minor product.

The interaction of catechol Fc-L-CatH_2_ with tin(IV) halides Ph_2_SnCl_2_ and SnCl_4_ in a toluene solution in the presence of a base (triethylamine) yields only ionic complexes (Fc-LH-Cat)SnPh_2_Cl, (Fc-LH-Cat)_2_SnCl_2_, and (Fc-LH-Cat)_2_SnPh_2_, where the Cat group is connected with ferrocenyl through the positively charged linker –CH=NH^+^–N=CH– (Scheme 17). These complexes also undergo electrochemical oxidation in three stages involving oxidation of catecholato centre, and ferrocenyl group. The –CH=NH^+^–N=CH– linker plays a role of the redox centre of the third type, which can be reduced in two stages (at the range of −1.7 to −2.2V vs. Cp_2_Fe/Cp_2_Fe^+^).

**Scheme 18:**
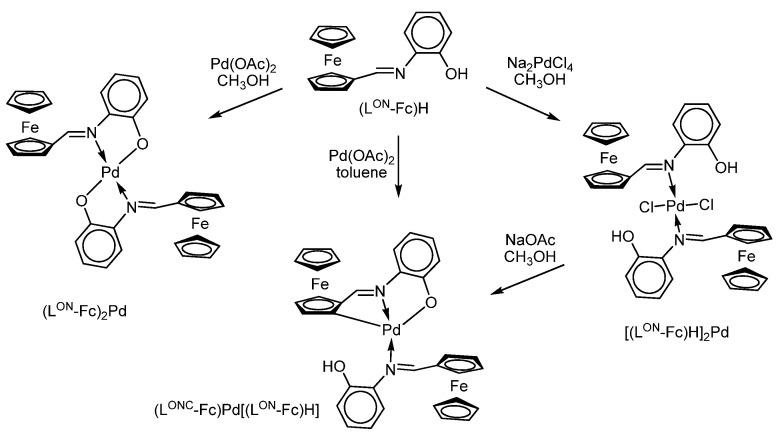
Different types of binding of ferrocene-containing o-iminophenol in reactions with palladium salts.

C. López et al. have found that ferrocenyliminophenol (L^ON^-Fc)H can exhibit an atypical nature of binding to the metal centre in reactions with Na_2_[PdCl_4_] and Pd(OAc)_2_ (Scheme 18) [91]. Thus, it was shown that iminophenol can act as a neutral donor ligand in reactions, being coordinated to the metal centre by the nitrogen atom of the imino-group (N) in complex [(L^ON^-Fc)H]_2_Pd, and as a bidentate one forming bis-iminophenolate derivative (L^ON^-Fc)_2_Pd containing N,O-chelating cycles. Alongside this, this iminophenol can form palladium complexes (L^ONC^-Fc)Pd[(L^ON^-Fc)H] with tridentate form of Schiff base ligand with two types of binding (N, and [C(sp^2^, ferrocene), N,O]^2-^) in reactions with Pd(OAc)_2_ in toluene (Scheme 18). The same authors have shown that this ferrocenyliminophenol behaves as a mono-, bi- or tridentate ligand in related platinum(II) complexes depending on the acid/base addition [110].

**Scheme 19:**
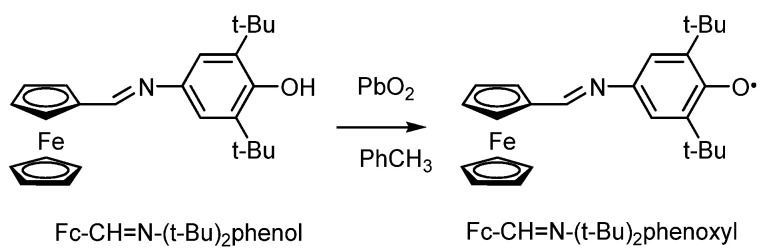
Oxidation of ferrocenyl-containing sterically hindered p-iminophenol to phenoxyl.

Another example of the ferrocene-containing Schiff base ligands are the N-(3,5-di-tert-butyl-4-hydroxyphenyl/benzyl)iminomethyl derivatives of ferrocene [99]. This work shows that the formation of the corresponding phenoxyl radicals takes place during the oxidation of phenolic derivatives Fc-CH=N-(t-Bu)_2_phenol with lead(IV) oxide in toluene (Scheme 19).

The hyperfine structure of the EPR spectrum of the ferrocene-containing N-(3,5-di-tert-butyl)-4-phenoxy radical corresponds to the HFC of an unpaired electron with two equivalent protons in the meta-positions of the phenoxy ring (a_i_(2 ^1^H) = 1.0 G) and the nitrogen atom of the imine-group (a_i_(^14^N) = 5.9 G). The introduction of an electron-donor ferrocenyl fragment into the para-position of the phenyl ring leads to an increase in the stability of the radical.

**Scheme 20:**
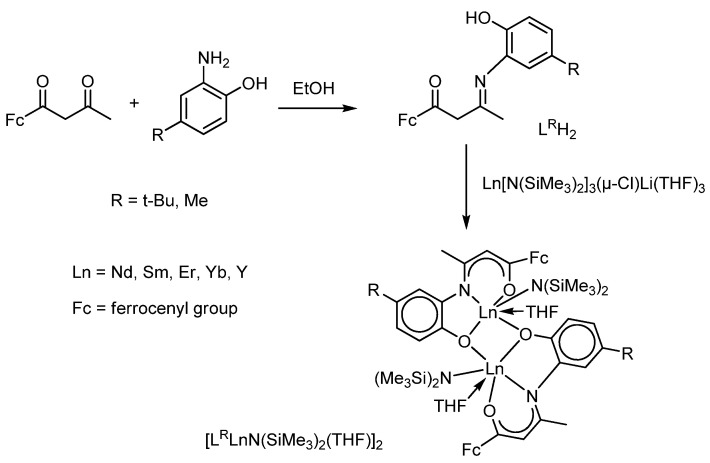
Preparation of lanthanide(III) silylamides stabilized by a ferrocene-containing β-ketoiminate ligand.

The ferrocene substituent can also be linked to the metal centre via the β-ketoiminate ligand [111,112] which, entering into the elimination reaction with lanthanide silylamides, forms the corresponding complexes [L^R^LnN(SiMe_3_)_2_(THF)]_2_ (Scheme 20). The redox properties of these complexes have not been studied. Still, the authors found that such lanthanide complexes, stabilized by a ferrocene-containing β-ketoiminate ligand, L^R^H_2_, can initiate ring-opening during the polymerization of ε-caprolactone and give polymers with a high molecular weight and a broad mass distribution.

However, a reaction of the ferrocene-containing β-ketoiminate ligand L^Me^H_2_ with the lanthanum derivative La[N(SiMe_3_)_2_]_3_(μ-Cl)Li(THF)_3_ in a 1:1 molar ratio resulted in an unexpected lanthanum–lithium bimetallic cluster [L^Me^_2_La{μ-Li(THF)}_2_(μ-Cl)]_2_, instead of the desired lanthanum amido complex [L^Me^LaN(SiMe_3_)_2_(THF)]_2_ [112]. The authors have supposed that the amine elimination reactions cause differences in the complexes structures due to the difference in the ionic radii of the lanthanide metals.

**Scheme 21:**
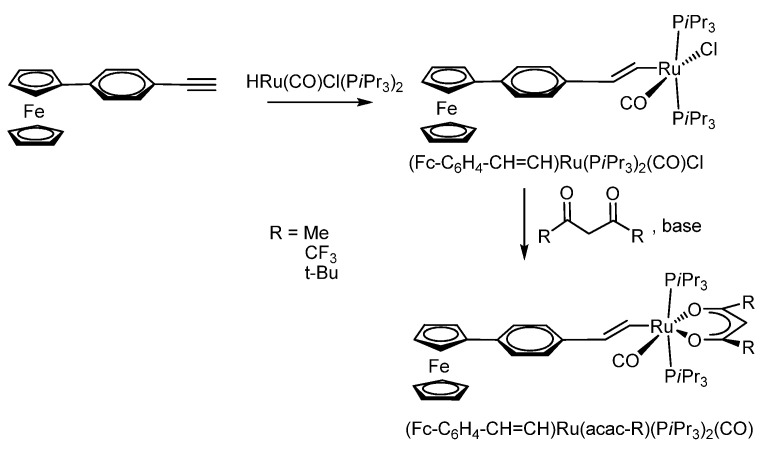
Synthesis of acetylacetonate ferrocenyl–styrylruthenium complexes of the type (Fc-C_6_H_4_-CH=CH)Ru(acac-R)(P*i*Pr_3_)_2_(CO) (where R = Me, CF_3_, t-Bu).

In the work of R.F. Winter et al. [113], a vinylphenyl substituent acts as a bridge connecting ferrocene with a metal centre. The acetylacetonate ferrocenyl–styrylruthenium complexes (Fc-C_6_H_4_-CH=CH)Ru(acac-R)(P*i*Pr_3_)_2_(CO) (Scheme 21) demonstrated the electron density distribution on the alkenyl-ruthenium fragment, depending on the substituent in acetylacetonate, which is reflected in the results of cyclic voltammetry. All ferrocene-containing complexes undergo two successive reversible one-electron oxidation stages, the potential of which depends on the substituents in the acetylacetonate ligand.

In the case of the complex (Fc-C_6_H_4_-CH=CH)Ru(acac)(P*i*Pr_3_)_2_(CO) based on an parent acetylacetate (acac) ligand (R = Me, Scheme 21), the authors have found that the oxidation of this compound leads to the formation of a radical cation, which can exist in the form of two valence tautomeric forms: Fc^+^C_6_H_4_-CH=CH{Ru^acac^} ↔ Fc-C_6_H_4_CH=CH{Ru^acac^}^+^, where {Ru^acac^} = Ru(acac)(P*i*Pr_3_)_2_(CO).

The positive charge in the tautomeric forms is localized either on the ferrocenyl centre or delocalized over the styrylruthenium fragment. The authors also found an interesting temperature dependence of the existence of tautomeric forms of the considered radical cation obtained by oxidation of complex (Fc-C_6_H_4_-CH=CH)Ru(acac)(P*i*Pr_3_)_2_(CO) using [Cp_2_Fe]^+^[PF_6_]^−^. The intensity of the isotropic signal (g_i_ = 2.0542) in the EPR spectrum gradually decreases at the decreasing temperature until it almost disappears at −120 °C. This fact indicates that the valence tautomer Fc^+^C_6_H_4_-CH=CH{Ru^acac^} is more thermodynamically preferable than the form Fc-C_6_H_4_CH=CH{Ru^acac^}^+^, which becomes predominant with increasing temperature.

**Scheme 22:**
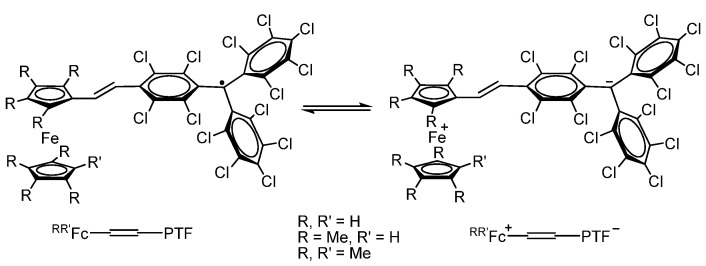
Electron transfer in a series of compounds ^RR’^Fc-CH=CH-PTF based on PTF radical.

J. Veciana et al. [114,115,116,117] have carried out a detailed analysis of the physicochemical properties for a series of compounds ^RR’^Fc-CH=CH-PTF based on the perchlorotriphenylmethyl radical (PTF) which acts as an electron acceptor bound through a vinylene π-bridge with various ferrocene derivatives (an electron donor) (Scheme 22).

There is an intense band at 387 nm in the electronic absorption spectra of radicals ^RR’^Fc-CH=CH-PTF, which was assigned to the radical of the chromophore (C_6_Cl_5_)_3_C^•^, as well as intense broad bands in the near-infrared region in the region of 800–2000 nm, which are caused by the intramolecular process electron transfer (IET process) from the donor ferrocenyl group to the acceptor radical group PTF. The replacement of hydrogen atoms in the ferrocenyl substituent by methyl groups has a significant effect on the energy of the IET process and leads to a red shift of the absorption band. The authors attribute this effect to enhancing the donor nature of the ferrocene fragments for methyl-substituted ferrocenyl derivatives, which reduces the energy of the IET process. An increase in the donor character of substituents in ferrocenyl was also reflected in the results of electrochemical studies. Thus, the oxidation of the Fc fragments is much easier in radicals ^R’^Fc-CH=CH-PTF, R = Me, R’ = H (+0.22 V) and R = R’ = Me (+0.09 V) than in Fc-CH=CH-PTF (+0.59 V) due to additional electron-donating methyl groups in cyclopentadienyl (Cp) rings.

**Scheme 23:**
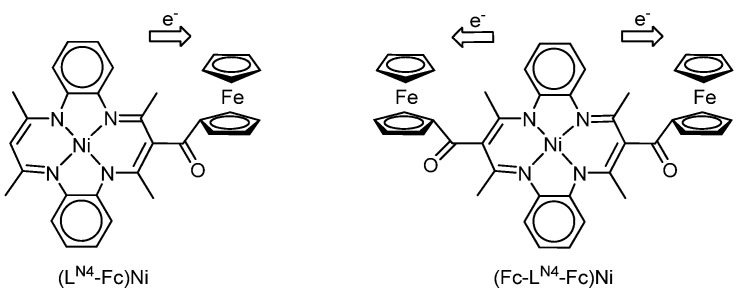
Mono- and bis-ferrocene-macrocyclic nickel complexes (L^N4^-Fc)Ni and (Fc-L^N4^-Fc)Ni.

Intriguing results were obtained by X. Shen, K. Sakata et al. [118]. They found that ferrocene substituents can exhibit the properties of electron acceptors under the conditions of electrochemical oxidation of ferrocene-containing 14-member macrocyclic nickel complexes (L^N4^-Fc)Ni and (Fc-L^N4^-Fc)Ni (Scheme 23).

The authors made these conclusions based on the difference between the first oxidation potentials (∆E^1^_pa_ = −0.05 and −0.06 V) of complexes (L^N4^-Fc)Ni and (Fc-L^N4^-Fc)Ni (E^1^_pa_ = 0.85 and 0.84 V, respectively) and the oxidation potential of free acetylferrocene CH_3_C(O)Fc (E^1^_pa_ = 0.90 V), where ∆E^1^_pa_= E^1^_pa_((L^N4^-Fc)Ni, (Fc-L^N4^-Fc)Ni)–E_pa_(CH_3_C(O)Fc). The authors attribute the negative values of ∆E^1^_pa_ for the complexes to the fact that electron transfer occurs from the π-conjugated system of the 14-membered ring to ferrocenes (Scheme 21), which confirms the fact that ferrocenes can act as electron acceptors.

**Scheme 24:**
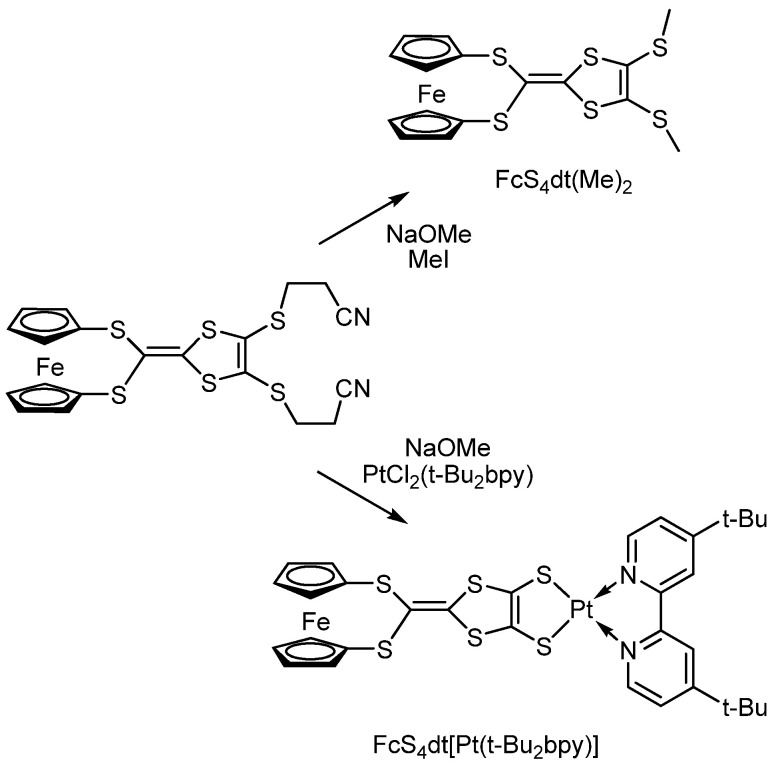
Synthesis of platinum dithiolate complex bearing the ferrocene group.

H. Nishihara et al. [119] have synthesized platinum dithiolate complexes bearing the ferrocene group bonded to a planar conjugated redox-active dithiolene fragment (Scheme 24).

The results of CV, EPR, and electron absorption spectroscopy indicate that the ferrocene fragment in FcS_4_dt(Me)_2_ undergoes oxidation first, but in FcS_4_dt[Pt(t-Bu_2_bpy)], dithiolene undergoes oxidation first. The anisotropic EPR spectrum of [FcS_4_dt(Me)_2_]^•+^—a product of the FcS_4_dt(Me)_2_ oxidation with [(4-BrC_6_H_4_)_3_N]^+^SbCl_6_^−^—in a frozen methylene chloride matrix has an axial symmetry with g_||_ = 3.70 and g_⊥_ = 1.70 pointing to the formation of ferrocenium derivatives.

The authors explained the appearance of a broad band at 2500 nm in the electronic absorption spectrum of the radical cation [FcS_4_dt(Me)_2_]^•+^ with the process of electron transfer from a donor (dithiolene fragment) to an acceptor (ferrocenium cation). While the broad band at 2000 nm for the oxidized form of the platinum complex [FcS_4_dt[Pt(t-Bu_2_bpy)]]^•+^ corresponds to the process of charge transfer from ferrocene to the dithiolate substituent. In the latter case, the dithiolene centre acts as an electron acceptor. In contrast, the ferrocene centre behaves as an electron donor, which indicates a significant electronic interaction between the ferrocene and dithiolene fragments.

Although the great interest in charge-transfer complexes based on metallocenes and the variety of scientific material collected to date on o-benzoquinone/o-iminobenzoquinone complexes, only a few examples of complexes of various metals are known that simultaneously contain both redox-active sites: o-benzoquinone/o-iminoquinone and ferrocene [120,121,122,123,124,125,126,127].

**Scheme 25:**
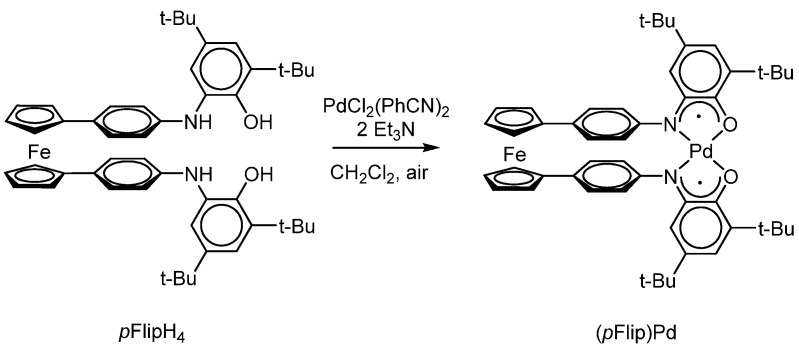
Palladium(II) bis-o-iminosemiquinolate, (*p*Flip)Pd, prepared by the exchange reaction of palladium(II) chloride with a ferrocene-containing bis-o-aminophenol *p*FlipH_4_.

S.N. Brown et al. [120] have synthesized a palladium bis-o-iminosemiquinolate complex (*p*Flip)Pd using the exchange reaction of palladium(II) chloride with a ferrocene-containing bis-o-aminophenol *p*FlipH_4_ in the presence of triethylamine on air (Scheme 25).

The data of electrochemical studies, as well as optical spectroscopy (titration using an oxidizer [CpFe(C_5_H_4_COCH_3_)]^+^[PF_6_]^−^ to the formation of a form [(*p*Flip)Pd]^+^) showed that the first oxidation involves that o-iminosemiquinolate centre, the interaction of which with the ferrocenyl substituent is minimal.

**Scheme 26:**
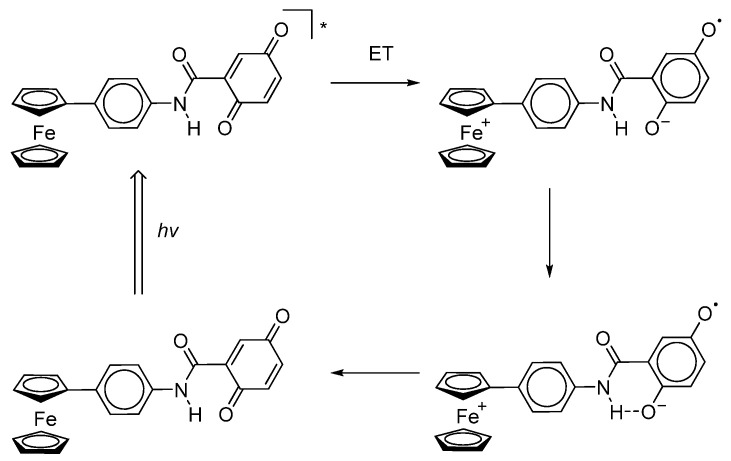
Photoinduced intramolecular electron transfer in p-benzoquinone bound with ferrocenyl through an amide bridge.

The first example of photoinduced intramolecular electron transfer was found in systems based on ferrocene bound to p-benzoquinone through an amide bridge in the meta-position (Scheme 26) [128].

As proof of single-electron intramolecular transfer, the authors of [128] referred to data from EPR spectroscopy and electron absorption spectroscopy. The HFS of the signal in the EPR spectrum of Fc^+^-Q^•−^, obtained by irradiating Fc-Q under laser flash photolysis conditions, is due to the HFC of an unpaired electron with three non-equivalent protons of the p-benzosemiquinone fragment with the following spectral parameters: g_i_ = 2.0055 and a_i_(^1^H) = 4.6, 2.05, and 1.75 G.

**Scheme 27:**
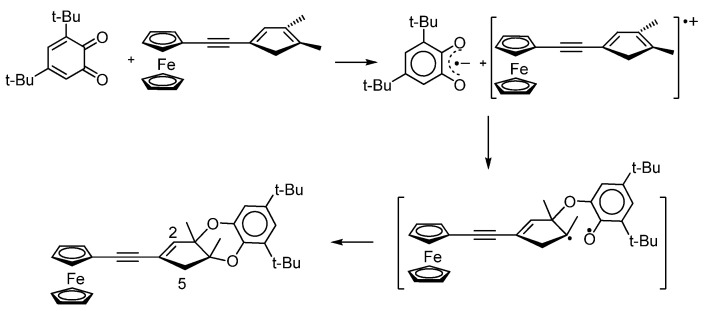
Heterocyclization of ferrocenyl-acetylene-cyclopentadiene with o-quinone.

When considering the formation mechanism of ferrocene-containing benzodioxines, the authors have found that 3,5-di-tert-butyl-o-benzoquinone, acting as an electron acceptor, can initiate the one-electron transfer reaction (Scheme 27) [123].

**Scheme 28:**
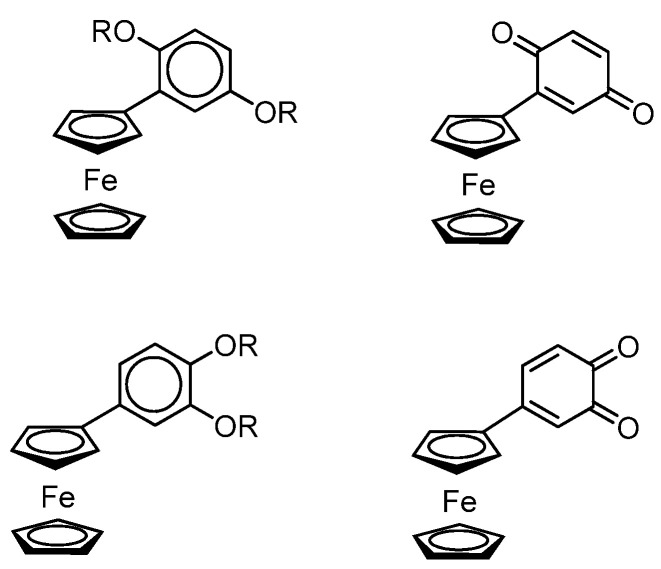
Ferrocenyl-substituted o-, p-quinones and hyrdoquinone-ethers.

Using EPR spectroscopy, the authors found that the interaction of o-benzoquinone with a ferrocene-containing diene in toluene at −20 °C leads to the formation of the anion radical o-benzosemiquinone (g = 2.0047, a_i_(^1^H) = 2.3 G). When this reaction is performed under conditions of an o-benzoquinone deficiency, it is possible to observe the EPR spectrum (g = 2.0037) of the radical cation, the HFS of which, according to the authors, is caused by the HFC of an unpaired electron with three groups of protons: (η^5^-C_5_H_4_Fe), C(5)–2H and C(2)–H (with a_i_(^1^H) = 5.8, 2.85, and 1.35 G, respectively) (the carbon atom numbering is shown in Scheme 27).A detailed electrochemical and spectroelectrochemical investigation of the redox properties of ferrocenyl-(hydro)-benzoquinones was carried out by the authors of the work [124] (Scheme 28).

The authors have found an intramolecular charge transfer from the ferrocene centre to the quinone part in ferrocenyl-benzoquinones, which is confirmed by a broad band (with a maximum at 713 nm) in the visible region of the electronic absorption spectrum. The authors also found that the one-electron reduction of Fc-SQ^•−^ or the one-electron oxidation to Fc^+^-Q leads to the disappearance of this transfer band with a change in the colour of the solution from intense green to pale yellow.

**Scheme 29:**
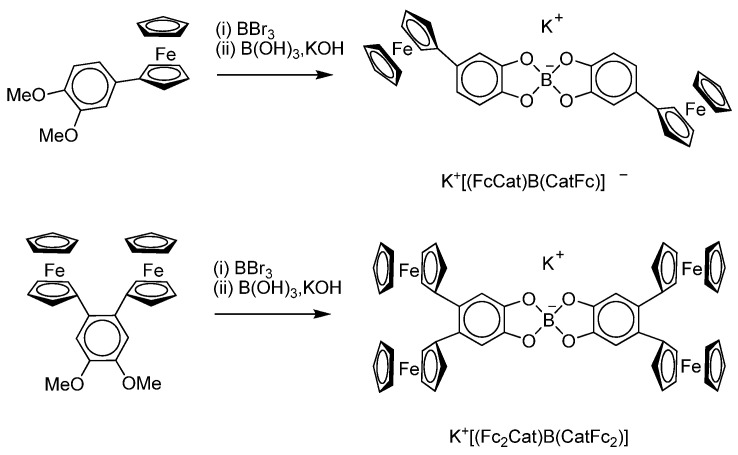
Synthesis of di- and tetraferrocenyl substituted potassium bis-catecholborates.

J. Kikuchi et al. [126] have synthesized di- and tetraferrocenyl substituted potassium bis-catecholborate complexes (Scheme 29) and investigated their redox behaviour.

Electrochemical investigations have shown that the spiroborate linkage between the mono- and di-ferrocenyl units led to electronic interactions over the boron centre: K^+^[(FcCat)B(CatFc)]^−^ displayed two one-electron oxidation processes at 0.376 and 0.678 V (vs. Cp_2_Fe/Cp_2_Fe^+^), while K^+^[(Fc_2_Cat)B(CatFc_2_)]^−^ exhibited four one-electron-oxidation waves at the range of 0.36–0.68 V in DMF at the potential switch from −0.15 to 0.85 V. All these waves are ferrocenyl-centred processes. The related bis-catecholate K^+^[(Cat)_2_B]^−^ without ferrocenyl groups undergoes oxidation waves at more positive potentials > 1.0 V assigned to the oxidation of catecholates.

**Scheme 30:**
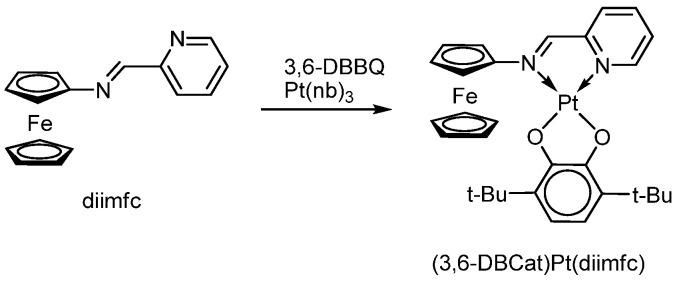
Reaction of platinum(0) tris-norbornene with 3,6-di-tert-butyl-o-benzoquinone in the presence of diimfc.

K. Heinze and S. Reinhardt described a Pt(II) catecholate complex (3,6-DBCat)Pt(diimfc) with a neutral-donor ferrocene-containing o-iminopyridine ligand (Scheme 30) [127].

The complex was synthesized by the reaction of the o-iminopyridine ligand with 3,6-di-tert-butyl-o-benzoquinone in the presence of Pt(nb)_3_, where nb is norbornene. The one-electron oxidation of the platinum catecholate obtained as a result of this interaction produces a cationic particle [(3,6-DBSQ)Pt(diimfc)]^+^ which contains the radical anion o-benzosemiquinone ligand. The HFS in the EPR spectrum of the formed radical complex is due to the HFC of an unpaired electron with two protons of the o-benzosemiquinone ligand 3,6-DBSQ (a_i_(2 ^1^H) = 4.25 G), as well as a platinum nucleus (a_i_(^195^Pt) = 24.2 G) and two nitrogen nuclei of the iminopyridine ligand diimfc (a_i_(2 ^14^N) = 1.75 G).

**Scheme 31:**
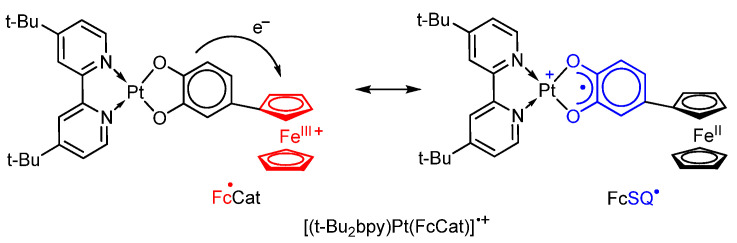
Electron delocalization between catecholate and Fc group in platinum(II) complex.

K.Tahara, J. Kikuchi et al. [125] found that the chemical one-electron oxidation of the platinum(II) complex (t-Bu_2_bpy)Pt(FcCat), in which the catecholate fragment is directly bonded to the ferrocenyl substituent at position four of the six-membered ring, with magic blue leads to the formation of the radical cation [(t-Bu_2_bpy)Pt(FcCat)]^•+^. The authors have shown a significant interaction between two redox-active centres, and an electron delocalization occurs between the catecholate fragment and the ferrocenium cation in such radical (Scheme 31). The form Fc^•^Cat contains ferrocenium cation and dianion catecholate. The electron transfer from the latter to Fc^+^ causes the oxidation of catecholate to radical anion o-semiquinone and reduction of Fc^+^ to Fc in the form FcSQ^•^.

This fact is confirmed by the presence of a wide charge-transfer band in the 1000 nm region in the electronic absorption spectra, and a band in the visible region (with a maximum at 594 nm) which corresponds to the ferrocenium cation.

Different interesting applications may be found during the investigations of such complexes bearing different types of redox-active moieties. Some examples are given below.

A series of titanium oxo clusters, Ti_3_O(OiPr)_6_(Cat)(FcCOO)_2_, Ti_7_O_4_(OiPr)_8_(Cat)_5_(FcCOO)_2_, and Ti_7_O_3_(OiPr)_12_(Cat)_4_(o-BDC), containing ferrocene-1-carboxylate (FcCOO^−^), catecholate (Cat^2−^), and o-benzene dicarboxylate (o-BDC), were found to be promising precursors for the efficient photovoltaic materials [129]. Q.-Y. Zhu, J. Dai et al. have shown that charge transfer occurs from ferrocenyl-containing ligands and catecholate to the titanium oxo cluster core, and the contribution of redox-ctive FcCOO^−^ is greater than that of Cat^2−^. The redox-active fragments demonstrate a synergistic effect on the enhancement of photocurrent responses due to the electron interaction between the neighbouring FcCOO^−^ and Cat^2−^ groups.

**Scheme 32:**
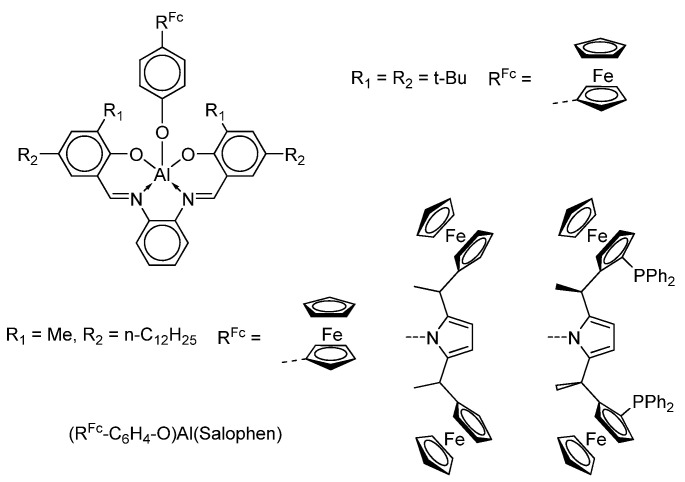
Ferrocenyl-containing aluminium Salophen complexes.

Authors have reported the synthesis of air-stable (R^Fc^-C_6_H_4_O)Al(Salophen) complexes (Scheme 32) which can undergo spontaneous self-assembly at the graphite/solution interface, forming highly-ordered nanostructures [130].

The redox properties of these derivatives functionalized with ferrocene units were studied by cyclic voltammetry in solution and on the surface. It was shown that these films have remarkable stability under the conditions used in the voltametric experiments (the reversible behaviour was observed after several cycles). Thus, the authors concluded that this synthetic strategy may be applied to introduce multiple functionalities with subnanometer precision at surfaces, to form ordered functional patterns. 

**Scheme 33:**
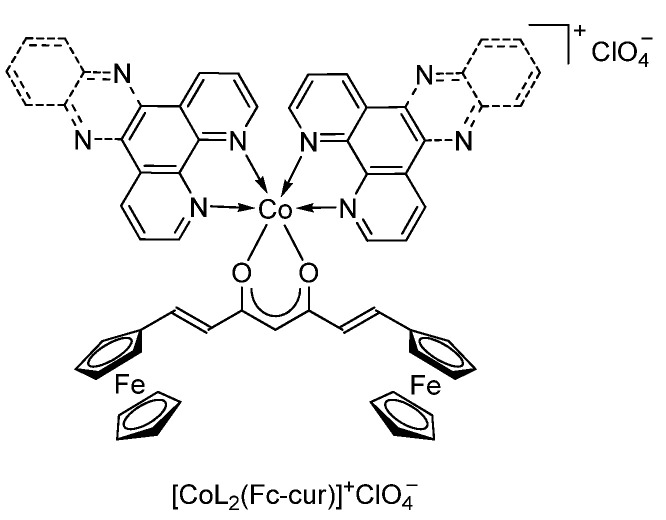
Complexes [CoL_2_(Fc-cur)]^+^ClO_4_^−^ based on a ferrocene-based curcuminoid ligand Fc-curH.

T.K. Goswami et al. [131] have synthesized cobalt(II) complexes [CoL_2_(Fc-cur)]^+^ClO_4_^−^ (Scheme 33) bearing a ferrocene-based curcuminoid (Fc-curH) ligand (L is 1,10-phenanthroline (phen) and dipyrido [3,2-a:2′,3′-c]phenazine (dppz)) and investigated their potential as photochemotherapeutic agents in vitro. These Co(II) complexes were found to be remarkably stable at physiological conditions with higher lipophilicity when compared to their cobalt(II) curcumin analogues.

The authors have shown that the ferrocenyl-containing curcuminoid cobalt(II) complexes can be considered excellent photochemotherapeutic agents in human cervical and lung cancer cells. The primary cell death mechanism was proposed to be apoptotic induced by light-assisted generation of reactive oxygen species.

## 4. Conclusions

Thus, the first part of the review contains the primary material describing the reactions of metallocenes, mainly ferrocene, with complexes based on various types of redox-active ligands. An analysis of the literature showed that the central part of the research is devoted to the study of the molecular structure and magnetic properties of ionic-type complexes formed in the course of such reactions, namely, ferrocenium salts and their derivatives containing, in addition to the paramagnetic ferrocenium cation, a diamagnetic or paramagnetic anion. Ferrocenium cations act as paramagnetic centres and can enter into intra- and intermolecular magnetic exchanges.

In addition, ferrocene is capable of effective electronic interaction with other π-systems (TCNE, TCNQ, bis-amidophenolates, etc.), demonstrating the ordering of structural units (D and A), which also makes a significant contribution to the nature of the magnetic exchange, while the Fe(II)/Fe(III) pair is stable and reversible. Among the accumulated material on this topic, ferrocene derivatives and ferrocene-containing metal complexes based on redox-active quinone-type ligands are represented insignificantly. The first example of charge-transfer complexes among non-transition metal compounds are complexes based on tin(IV) halide-o-semiquinolates and ferrocene. The dependence of magnetic properties both on the nature of the auxiliary substituents at the central tin atom (halogens) and from the solvent was shown for these complexes.

The second part of the review covers the metal complexes which simultaneously combine in their composition, in addition to the ferrocene substituent, additional redox-active centres, such as quinone- and dithiolene-type ligands, and other substituents covalently bonded to ferrocene or coordinated to the central metal atom in a complex containing a ferrocene fragment in an auxiliary ligand. Such complexes have a wide range of redox transitions, which is determined not only by the nature of the substituents in the redox-active ligand but also by the substituents in the ferrocene fragment, as well as by the nature of the bridging group (if any) linking two different redox-active centres. An analysis of the literature data on the coordination chemistry of redox-active ligands with ferrocenes has shown that one of the most common methods for the functionalization of complexes of various metals with ferrocene groups is the use of o-iminophenol ligands such as Schiff bases. Also, β-ketoiminate ligands, vinylphenyl substituents, etc., have received considerable distribution as a bridge that binds ferrocene. Among the coordination compounds with redox-active ligands, there are also several examples of heteroligand derivatives containing both o-benzoquinone/iminoquinone and ferrocene centres in their composition. Further study of such model systems will provide information on the influence of various factors on redox processes, including the electron transfer process, which is a fundamental stage of many chemical reactions and is of crucial importance in many catalytic and biochemical processes, including photosynthesis, respiration, the transmission of nerve impulses, etc.

## Data Availability

Not applicable.

## Appendix A

**Table A1:** The complexes abbreviations and references.

Complex	Abbreviation Description	Page	Ref.
[Cp*_2_Fe]^•+^[TCNE]^•−^ [Cp*_2_Fe]^•+^[TCNQ]^•−^	TCNE—tetracyanoethylene; TCNQ—tetracyanoquinodimethane	2	[62,67]
[L^1^]^+^[Ni(mnt)_2_]^−^ [L^1^]^+^[Pt(mnt)_2_]^−^ [L^2^]^+^[Ni(mnt)_2_]^−^ [L^3^]^+^[Ni(mnt)_2_]^−^	mnt—maleonitriledithiolate ligand; L^1^, L^2^, L^3^—different thioether-, S-heterocyclic-, or vinyl tetrathiafulvalene-substituted 2,2′,3,3′,4,4′-hexamethylferrocenes	3	[68]
[Cp*_2_M]^+^[Co(HMPA-B)]^−^, M = Co, Fe	HMPA-B—bis(2-hydroxy-2-methylpropanamido)benzene	3	[69]
[Ni^III^(3,6-DBCat)_2_]^2^[Cp_2_Co]^+^	3,6-DBCat—3,6-di-tert-butylcatecholate	3	[70]
[Cp_2_M]^+^[Cr^III^(X_4_SQ)_2_(X_4_Cat)]^+^, M = Co, Fe [Cp_2_Co]^+^_2_[Cr^III^(X_4_SQ)(X_4_Cat)_2_]^2+^	X_4_SQ—tetrahalogeno-o-benzosemiquinone, X_4_Cat—tetrahalogenocatecholate; X = Cl, Br	4	[71,72]
[Fe(NO)(L)_2_]^−^[Cp_2_Co]^+^ [Fe(NO)(L)_2_]^2−^[Cp_2_Co]^+^_2_	L—1,2-diarylethene-1,2-dithiolate, aryl—is phenyl, *p*-tolyl, *p*-methoxyphenyl	4	[73]
(Q-CH_2_CH_2_-SQ)^•−^[Cp_2_Co]^+^ (Q-CH_2_CH_2_-SQ)Cu^I^(dppfc)	Q—3,6-di-tert-butyl-1,2-benzoquinone-4-yl, SQ—its radical-anion o-benzosemiquinone	5	[74]
(3,6-DBSQ)Cu^I^(dppfc)	3,6-DBSQ—3,6-di-tert-butyl-o-benzosemiquinone	5	[75]
Ph_3_Sb(Cat-SQ)Cu^I^(dppfc)	Cat-SQ—6-tert-butyl-4-(6-tert-butyl-3-methyl-1,2-benzosemiquinone-4-yl)-3-methylcatecholate	5	[76]
(Q-TTF-SQ)Cu^I^(dppfc)	Q-TTF-SQ—monoreduced (o-semiquinone) derivative of bis-o-benzoquinone with tetrathiafulvalene linker	5	[77]
[(3,6-DBCat)SnBr_3_(THF)]^–^[Cp_2_Fe]^+^ [(3,6-DBSQ)(3,6-DBCat)SnCl_2_]^–^[Cp_2_Fe]^+^	3,6-DBCat—3,6-di-tert-butylcatecholate; 3,6-DBSQ—3,6-di-tert-butyl-o-benzosemiquinone	5	[78]
U(salfen)_2_ K_3_[U(bis-salfen)(Hbis-salfen)] K_4_[U(Hbis-salfen)_2_]	salfen—N,N′-bis-(salicylidene)-1,1′-diaminoferrocene; bis-salfen and Hbis-salfen—products of an intramolecular C-C bond formation between two imino groups of salfen	6	[86]
(L^ONO^-Fc)M^II^∙Py, M = Ni, Cu (L^ONO^-Fc)M^II^∙(4,4′-bipy)∙M^II^(L^ONO^-Fc), M = Ni, Cu	L^ONO^—doubly deprotonated dianion of 2-((4-ferrocenyl-4-hydroxybut-3-en-2-ylidene)amino)phenol	7	[87,89]
(L^ONO^-Fc)M^II^∙(PyMP), M = Ni, Cu	PyMP—pyridyl 2,6-diphenylmethylenepyran	7	[90]
(L^ONNO1^-Fc)M, M = Ni, Cu [(Cp*Ru^+^L^ONNO1^-Fc)M]PF_6_^−^, M = Ni, Cu (L^ONNO2^-Fc)Ni [(Cp*Ru^+^L^ONNO2^-Fc)Ni]PF_6_^−^	L^ONNOn^-Fc (n = 1,2)—unsymmetrical Schiff base ligands—derivatives of condensation products of 3-(2-aminoethyl(or phenyl)imino)-1-ferrocenylbut-1-en-1-ol and 5-(Br, H, or OH)-substituted salicylaldehyde	8	[93,94,95]
(L^ONNO^-Fe)M, M = Ni, Cu	L^ONNO^-Fe—the unsymmetrical Schiff base ligands from some other substituted salicylaldehydes	9	[92,96]
[(L^ONO^-Fc)_2_Co]^−^[K(EtOH)_2_]^+^	L^ONO^—doubly deprotonated dianion of 2-((4-ferrocenyl-4-hydroxybut-3-en-2-ylidene)amino)phenol	9	[102]
(L^SN^-Fc)_2_M, M = Ni, Zn, Pd	L^SN^-Fc—2-(ferrocenylmethyleneamino)-thiophenolate	9	[97,98]
(^R^L^ON^-Fc)_2_Sn^II^	^R^L^ON^-Fc—functionalized 2-(ferrocenylmethyleneamino)phenolate	10	[103]
(^R^L^ON^-Fc)_2_M^II^, M = Co, Ni (^R1R2^L^ON^-Fc)_2_M^II^, M = Ni, Cu (^R^L^ON^-Fc)_2_Co^III^(3,6-DBSQ)	^R1R2^L^ON^-Fc—bifunctionalized 2-(ferrocenyl-methyleneamino)phenolate	10	[104,105]
(^R^L^ON^-Fc)_2_Sn^IV^(R-Cat) (^R^L^ON^-Fc)_2_Sn^IV^(AP)	R-Cat—substituted catecholate; AP—N-aryl-4,6-di-tert-butyl-o-amidophenolate	11	[106,107]
(L^ON^-Fe)_2_Mn∙4H_2_O (L^ON^-Fe)_2_VO∙H_2_O (L^ON^-Fe)Zn(NO_3_)∙3H_2_O (L^ON^-Fe)Pd(CH_3_COO)∙2H_2_O	L^ON^-Fc—2-(ferrocenylmethyleneamino)phenolate	11	[108]
(Fc-L-Cat)SbPh_3_ (Fc-LH-Cat)SbPh_3_Br (Fc-LH-Cat)SnPh_2_Cl (Fc-LH-Cat)_2_SnCl_2_ (Fc-LH-Cat)_2_SnPh_2_	Fc-L-Cat—3,5-di-tert-butylcatecholate bound in 6th position with Fc group via –CH=N–N=CH– linker; Fc-LH-Cat—3,5-di-tert-butylcatecholate bound in 6th position with Fc group via –CH=N^+^H–N=CH– linker	11- -12	[109]
[(L^ON^-Fc)H]_2_Pd (L^ON^-Fc)_2_Pd (L^ONC^-Fc)Pd[(L^ON^-Fc)H]	L^ON^-Fc—2-(ferrocenylmethyleneamino)phenolate; L^ONC^-Fc—tridentate ligand O,N,C-bound with Pd; (L^ON^-Fc)H—2-(ferrocenylmethyleneamino)phenol as monodentate N-donor ligand	12	[91]
Fc-CH=N-(t-Bu)_2_phenol	2,6-di-tert-butyl-4-(ferrocenylmethyleneamino)phenol	13	[99]
Fc-CH=N-(t-Bu)_2_phenoxyl	2,6-di-tert-butyl-4-(ferrocenylmethyleneamino)phenoxy radical		
[L^R^LnN(SiMe_3_)_2_(THF)]_2_, Ln = Ns, Sm, Er, Yb, Y; [L^Me^_2_La{μ-Li(THF)}_2_(μ-Cl)]_2_	L^R^—ferrocenyl-containing β-ketoiminate ligand	13- -14	[111,112]
(Fc-C_6_H_4_-CH=CH)Ru(acac-R)(P*i*Pr_3_)_2_(CO)	acac-R—disubstituted β-diketonate ligand	14	[113]
^RR’^Fc-CH=CH-PTF	PTF—perchlorotriphenylmethyl radical	15	[114,115,116,117]
(L^N4^-Fc)Ni (Fc-L^N4^-Fc)Ni	L^N4^-Fc and Fc-L^N4^-Fc—mono- and diferrocenyl-containing macrocyclic ligands based on dibenzo[1,4,8,11]tetraazacyclotetradecines	15	[118]
FcS_4_dt(Me)_2_ and FcS_4_dt[Pt(t-Bu_2_bpy)]	FcS_4_dt(Me)_2_—1,1′-ferrocenyl-containing redox-active dithiolene type ligand	16	[119]
(*p*Flip)Pd	*p*Flip—ferrocene-containing bis-o-aminophenol	16	[120]
ferrocenyl-amide-p-benzoquinone	-	17	[128]
ferrocenyl-acetylene-benzodioxines	-	17	[123]
ferrocenyl-(hydro)-benzoquinones	-	18	[124]
K^+^[(FcCat)B(CatFc)]^−^ K^+^[(Fc_2_Cat)B(CatFc_2_)]^−^	FcCat—4-ferrocenylcatecholate Fc_2_Cat—4,5-diferrocenylcatecholate	18	[126]
(3,6-DBCat)Pt(diimfc) [(3,6-DBSQ)Pt(diimfc)]^+^	diimfc—N-ferrocenyl-2-iminomethylpyridine	19	[127]
(t-Bu_2_bpy)Pt(FcCat), [(t-Bu_2_bpy)Pt(FcCat)]^•+^	t-Bu_2_bpy—4,4′-di-tert-butyl-2,2′-bipyridine FcCat—4-ferrocenylcatecholate	19	[125]
Ti_3_O(OiPr)_6_(Cat)(FcCOO)_2_ Ti_7_O_4_(OiPr)_8_(Cat)_5_(FcCOO)_2_ Ti_7_O_3_(OiPr)_12_(Cat)_4_(o-BDC)	FcCOO—ferrocene-1-yl-carboxylate, Cat—catecholate, o-BDC—o-benzene dicarboxylate	20	[129]
(R^Fc^-C_6_H_4_O)Al(Salophen)	R^Fc^—ferrocenyl, diferrocenylpyrrol, or bis-(diphenylphosphinoferrocenyl)pyrrol type groups; Salophen—N,N′-bis-(salicylidene)-1,2-diaminobenzene	20	[130]
[CoL_2_(Fc-cur)]^+^ClO_4_^−^	L—1,10-phenanthroline (phen) or dipyrido[3,2-a:2′,3′-c]phenazine (dppz); Fc-cur—ferrocene-based curcuminoid ligand	20	[131]
